# A Variant of the Autophagy-Related 5 Gene Is Associated with Child Cerebral Palsy

**DOI:** 10.3389/fncel.2017.00407

**Published:** 2017-12-18

**Authors:** Jianhua Xu, Lei Xia, Qing Shang, Jing Du, Dengna Zhu, Yangong Wang, Dan Bi, Juan Song, Caiyun Ma, Chao Gao, Xiaoli Zhang, Yanyan Sun, Liping Zhu, Xiaoyang Wang, Changlian Zhu, Qinghe Xing

**Affiliations:** ^1^Institute of Biomedical Science and Children's Hospital, and Key Laboratory of Reproduction Regulation of the National Population and Family Planning Commission (NPFPC), Shanghai Institute of Planned Parenthood Research (SIPPR), IRD, Fudan University, Shanghai, China; ^2^Henan Key Laboratory of Child Brain Injury, Department of Pediatrics, The 3rd Affiliated Hospital of Zhengzhou University, Zhengzhou, China; ^3^Department of Pediatrics, Henan Children's Hospital, Zhengzhou, China; ^4^Child Rehabilitation Center, The 3rd Affiliated Hospital of Zhengzhou University, Zhengzhou, China; ^5^Shanghai Center for Women and Children's Health, Shanghai, China; ^6^Perinatal Center, Sahlgrenska Academy, Gothenburg University, Gothenburg, Sweden; ^7^Center for Brain Repair and Rehabilitation, Sahlgrenska Academy, Gothenburg University, Gothenburg, Sweden

**Keywords:** ATG5, autophagy, cerebral palsy, ELISA, polymorphisms, single nucleotide

## Abstract

Cerebral palsy (CP) is a major cause of childhood disability in developed and developing countries, but the pathogenic mechanisms of CP development remain largely unknown. Autophagy is a highly conserved cellular self-digestion of damaged organelles and dysfunctional macromolecules. Growing evidence suggests that autophagy-related gene 5 (ATG5)-dependent autophagy is involved in neural development, neuronal differentiation, and neurological degenerative diseases. The aim of this study was to analyze *ATG5* protein expression and gene polymorphisms in Chinese patients with CP and to evaluate the importance of ATG5 in the development of CP. Five polymorphisms from different regions of the *ATG5* gene (rs510432, rs3804338, rs573775, rs2299863, and rs6568431) were analyzed in 715 CP patients and 658 controls using MassARRAY. Of these, 58 patients and 56 controls were selected for measurement of plasma ATG5 level using ELISA. The relevance of disease-associated SNPs was evaluated using the SHEsis program. We identified a significant association between rs6568431 and CP (OR = 1.388, 95% CI = 1.173~1.643, *P*_*allele*_ = 0.0005, *P*_*genotype*_ = 0.0015). Subgroup analysis showed a highly significant association of rs6568431 with spastic CP (n = 468, OR = 1.511, 95% CI = 1.251~1.824, *P*_*allele*_ = 8.50e^−005^, *P*_*genotype*_ = 1.57e^−004^) and spastic quadriplegia (OR = 1.927, 95% CI = 1.533~2.421, *P*_*allele*_ = 7.35e^−008^, *P*_*genotype*_ = 3.24e^−009^). Furthermore, mean plasma ATG5 levels were lower in CP patients than in controls, and individuals carrying the AA genotype of rs6568431 that was positively associated with CP had lower plasma ATG5 levels (*P* < 0.05). This study demonstrated an association of an *ATG5* gene variant and low level of ATG5 protein with CP, and stronger associations with severe clinical manifestations were identified. Our results provide novel evidence for a role of ATG5 in CP and shed light on the molecular mechanisms underlying this neurodevelopmental disorder.

## Introduction

Cerebral palsy (CP) is a group of permanent developmental disorders of movement and posture that appear in early childhood, often accompanied by epilepsy and disturbances in perception, sensation, cognition, behavior, and communication, as well as secondary musculoskeletal problems (Colver et al., 2014; Hoare, 2014). CP can cause activity limitation due to non-progressive disturbances in the developing fetal or infant brain (Colver et al., 2014). Though antenatal and perinatal care have improved, overall prevalence has remained stable at 2–3.5 cases per 1,000 live births in the past 40 years (Yeargin-Allsopp et al., 2008; Colver et al., 2014). CP is a major cause of childhood disability in developed and developing countries (Wu et al., 2011; Tatla et al., 2013), but the underlying pathogenic factors and molecular mechanisms for the development of CP remain largely unknown. Some recent studies have connected many cases of CP to genetic alterations that may directly cause or confer susceptibility to CP (McMichael et al., 2015; Bi et al., 2016; Fahey et al., 2017).

Autophagy is a highly regulated process involving the degradation of unused macromolecules and damaged cell organelles in the cytosol through the lysosomal system (Shintani and Klionsky, 2004; De Biase et al., 2017). Three forms of autophagy are commonly described: macroautophagy, microautophagy, and chaperone-mediated autophagy. In macroautophagy, targeted cytoplasmic constituents are isolated from the rest of the cell within a double-membraned vesicle known as an autophagosome. The autophagosome eventually fuses with lysosomes and the contents are degraded and recycled (Mizushima et al., 2011). Autophagy is involved in many physiological processes, including development, differentiation, aging, innate immunity, quality control of intracellular proteins and organelles, starvation adaptation, and tumor suppression (Oppenheim et al., 2008; Tasdemir et al., 2008; Wang et al., 2017). Furthermore, dysregulated autophagy has been implicated in inflammatory and neurodegenerative diseases (Levine and Kroemer, 2008; Wong and Cuervo, 2010; Deretic et al., 2013).

Autophagy-related gene 5 (ATG5), a key autophagy gene, contributes to autophagosome formation, and its deletion can effectively block autophagy in yeast, mammal cells, or mice (Kuma et al., 2004). In this process, ATG12, activated first by ATG7, is transferred to ATG10, and finally covalently attached to ATG5. The ATG12-ATG5 conjugate localizes to autophagosome precursors and dissociates just before or after completion of autophagic-vacuole formation (Mizushima et al., 2003, 2011). Furthermore, ATG5 may have its own autophagy-independent functions, i.e., isolated ATG functions, which involve gene-specific function instead of effectuation of the integral autophagy pathway. For instance, the ATG5-ATG12 conjugate acts as a suppressor of retinoic acid-inducible gene 1-like receptor (RLR) signaling (Jounai et al., 2007; Tal et al., 2009; Zhou and Zhang, 2012), ATG5 has macroautophagy-independent roles in controlling resistance to *Mycobacterium tuberculosis (Mtb)* infection *in vivo* (Jacqueline et al., 2015), and Atg5 protein participates in immunity and intracellular killing of pathogens via autophagosome-independent processes in phagocytic cells (Zhao et al., 2008).

Mice deficient in ATG5 die within 1 day of delivery (Kuma et al., 2004), while mice with specific deletion of ATG5 in neural cells can develop gradual defects in motor function accompanied by the accumulation of cytoplasmic inclusion bodies in neurons (Hara et al., 2006). ATG5 loss in Purkinje cells causes the cells to degenerate and animals to exhibit ataxic gait (Nishiyama et al., 2007). Elevated ATG5 expression, on the other hand, interferes with the neuronal differentiation of neuroblastoma cells (Chae et al., 2009), and ATG5 has been shown to function during early neuronal differentiation of stem and progenitor cells (Vázquez et al., 2012). Together, these data indicate that ATG5 is involved in neural development, neuronal differentiation, and neurological degenerative diseases.

Recent studies have shown that genetic variants of ATG5 play a role in predisposition to various neurodegenerative diseases such as Parkinson's disease (PD; Chen et al., 2013), autoimmune diseases such as systemic lupus erythematosus (SLE; López et al., 2013), and inflammatory diseases such as asthma (Martin et al., 2012). Although ATG5 is increasingly recognized for its involvement in the pathogenesis of CP, the association of ATG5 genetic variants with CP has not yet been investigated. Therefore, we analyzed five single nucleotide polymorphisms (SNPs) from different regions of the *ATG5* gene in 715 patients with CP and 658 controls. Furthermore, we used a plasma ATG5 protein assay to clarify the functional association of *ATG5* polymorphisms and CP, based on the reports that some polymorphisms within *ATG5* correlated with ATG5 expression (Zhou and Zhang, 2012; Zheng et al., 2015).

## Materials and methods

### Study subjects

A total of 715 patients with CP (30.63% girls and 69.37% boys, mean age: 18.26 ± 15.13 months old) were recruited from centers for CP rehabilitation in the 3rd Affiliated Hospital of Zhengzhou University and Henan Children's Hospital from July 1, 2010 to May 31, 2014. Another 658 controls (33.43% girls and 66.57% boys, mean age: 19.53 ± 17.18 months old) were chosen from a physical examination population from the same hospital during the same time period. Of these, 58 patients with CP (38 boys and 20 girls, 20.54 ± 13.13 months old) and 56 controls (42 boys and 14 girls, 21.95 ± 14.65 months old) were selected for plasma ATG5 ELISA based on the strict exclusion criteria. First, all children with cough, fever, acute respiratory illness, any other indications of infection, or dramatic changes of bodyweight within the past 3 months were excluded. Second, the CP patients who have received any other medication within 1 month were excluded. All recruited children were from the Han population of the Henan Province, and their parents were asked to sign informed consent. Approval for the study was obtained from the ethics committee of Zhengzhou University in accordance with the Helsinki declaration.

The clinical information database included sex, CP subtype, gestational age, birth weight, birth asphyxia, neonatal complications [periventricular leukomalacia (PVL), hypoxic-ischemic encephalopathy (HIE), and mental retardation (MR)], and maternal risk factors [premature rupture of membrane (PROM), threatening premature labor (TPL), and pregnancy-induced hypertension (PIH); Table 1]. Diagnosis and classification of CP and diagnosis of birth asphyxia, PVL, HIE, MR, PROM, PIH, and TPL were described previously (Bi et al., 2014).

**Table 1:** Clinical characteristics of all participants.

**Characteristic**	**CP cases**		**Control**	
	**Total (%)**	**M/F (*n*)**	**Total (%)**	**M/F (*n*)**
**GESTATIONAL AGE**				
Preterm (< 37 weeks)	40 (5.59)	33/7	11 (1.67)	10/1
Term (≥37 weeks)	675 (94.41)	463/212	647 (98.33)	428/219
Total	715 (100)	496/219	658 (100)	438/220
**BIRTH WEIGHT**				
<2,500 g	29 (4.06)	20/9	17 (2.58)	13/4
≥2,500 g	686 (95.94)	485/201	641 (97.42)	425/216
Total	715 (100)	505/210	658 (100)	438/220
**TYPE OF CP**				
Spastic CP	468 (65.45)	338/130	–	–
Non-spastic CP	247 (34.55)	158/89	–	–
Total	715 (100)	496/219	658 (100)	438/220
**BIRTH ASPHYXIA**				
Asphyxia	212 (29.65)	157/55	10 (1.52)	7/3
No asphyxia	503 (70.35)	339/164	648 (98.48)	431/217
Total	715 (100)	496/219	658 (100)	438/220
**COMPLICATION**				
CP with PVL	67 (9.37)	53/14	–	–
CP without PVL	648 (90.63)	445/203	–	–
CP with HIE	96 (13.45)	71/25	–	–
CP without HIE	619 (86.55)	421/198	–	–
CP with MR	289 (40.42)	199/90		
CP without MR	426 (59.58)	297/129		
**MATERNAL FACTORS**				
PROM	68 (9.51)	51/17	24 (3.65)	16/8
No PROM	647 (90.49)	425/202	634 (96.35)	420/214
TPL	57 (7.97)	40/17	33 (5.02)	27/6
No TPL	648 (92.03)	454/196	625 (94.98)	411/214
PIH	27 (3.78)	21/6	8 (1.22)	7/1
No PIH	688 (96.22)	476/212	650 (98.78)	431/219

*CP, cerebral palsy; M, male; F, female; PVL, periventricular leukomalacia; PROM, premature rupture of membrane; TPL, threatened premature labor; PIH, pregnancy-induced hypertension*.

### Selection of SNPs and genotyping

Venous blood samples were collected into tubes containing EDTA in the second morning after hospitalization. The samples were centrifuged at 1,500 × g for 20 min at 4°C, and plasma was aliquoted and stored at −80°C.

A total of five SNPs (rs510432, rs3804338, rs573775, rs2299863, and rs6568431) of the *ATG5* gene were selected from the dbSNP database (http://www.ncbi.nlm.nih.gov/SNP). The rs510432 SNP is located in the upstream region of the *ATG5* gene, while the other four SNPs are located in introns: rs3804338 (2nd), rs573775 (2nd), rs2299863 (7th), and rs6568431 (9th). The minor allele frequencies of these five SNPs in the Chinese Han population are more than 0.05.

Genomic DNA was isolated from white blood cells using a QIAamp DNA Blood Kit (QIAGEN, USA) according to the manufacturer's instructions. SNPs were genotyped using the Sequenom iPlex MassARRAY platform (Sequenom, San Diego, CA, USA) and blinded to the clinical status of the subjects.

### ELISA of plasma ATG5 levels

Before ELISA, frozen samples were removed from the freezer and allowed to reach room temperature (20–25°C) with gentle mixing. ATG5 levels in the plasma of 58 patients and 56 controls were measured using a Human ATG5 ELISA Kit (Cloud-Clone Corp., USA) according to the manufacturer's protocol. The absorbance at 405 nm was measured with a SpectraMax 190 Microplate Spectrophotometer (Molecular Devices, USA). The ATG5 level in plasma was normalized to the kit standard, and data were summarized as mean ± standard error of the mean (SEM).

### Statistical analysis

For gene analysis, the Hardy-Weinberg equilibrium (HWE), allele and genotype frequencies, odds ratio (OR), 95% confidence intervals (95% CI), linkage disequilibrium (LD), and haplotype frequencies were performed using the SHEsis online software platform (http://analysis.bio-x.cn/myAnalysis.php). Discrepancies of genotype and allele frequencies between the patients and controls were compared using the χ^2^ test. The multiple testing on each individual SNP was corrected using Bonferroni correction. Quantitative results were analyzed using Statistical Product and Service Solutions (SPSS) (version 20.0, SPSS Inc., Chicago, IL, USA). Differences were determined by Student's *t*-test; *P* < 0.05 was considered statistically significant.

## Results

### Overall analysis: allele, genotype, and haplotype frequency of *ATG5* polymorphisms

Genotype frequencies of the five polymorphisms of *ATG5* showed no significant deviations from HWE in controls (*p* > 0.05; Table 2). Among all patients with CP and controls, rs6568431 allele and genotype frequencies reached significance (OR = 1.388, 95% CI = 1.173~1.643, *P*_allele_ = 0.0005, *P*_*genotype*_ = 0.0015 after Bonferroni correction), and patients with CP had significantly higher A allele and AA genotype frequencies. These results showed that individuals carrying the rs6568431 AA genotype had a significantly higher risk of CP than those with the CC genotype.

**Table 2:** Allele and genotype frequencies of ATG5 genetic variants in CP (*n* = 715) and controls (*n* = 658).

	**Allele**	***P***	**OR** **(95%CI)**	**Genotype**	***P***	**Hardy-Weinberg equilibrium test**
**rs510432**	A	G			A/A	A/G	G/G		
CP	360 (0.384)	578 (0.616)	**0.285**	0.906	80 (0.171)	200 (0.426)	189 (0.403)	**0.324**	0.033
controls	414 (0.407)	602 (0.593)		(0.755~1.086)	88 (0.0.173)	238 (0.469)	182 (0.358)		0.502
**rs3804338**	C	T			C/C	C/T	T/T		
CP	1123 (0.867)	173 (0.133)	**0.968**	1.005	486 (0.750)	151 (0.233)	11 (0.017)	**0.894**	0.853
controls	982 (0.866)	152 (0.134)		(0.795~1.270)	423 (0.746)	136 (0.240)	8 (0.014)		0.429
**rs573775**	C	T			C/C	C/T	T/T		
CP	808 (0.650)	436 (0.350)	**0.477**	0.940	255 (0.410)	298 (0.479)	69 (0.111)	**0.316**	0.192
controls	747 (0.663)	379 (0.337)		(0.793~1.114)	251 (0.446)	245 (0.435)	67 (0.119)		0.544
**rs2299863**	G	T			G/G	G/T	T/T		
CP	79 (0.061)	1213 (0.939)	**0.979**	0.996	4 (0.006)	71 (0.110)	571 (0.884)	**0.764**	0.277
controls	70 (0.061)	1070 (0.939)		(0.714~1.388)	2 (0.004)	66 (0.116)	502 (0.881)		0.914
**rs6568431**	A	C			A/A	A/C	C/C		
CP	459 (0.388)	723 (0.612)	**0.0001**^a^	1.388	107 (0.181)	245 (0.415)	239 (0.404)	**0.0003**^b^	0.002
controls	381 (0.314)	833 (0.686)		(1.173~1.643)	63 (0.104)	255 (0.420)	289 (0.476)		0.545

*OR, odds ratio; CI, confidence interval*.

a *After Bonferroni correction, the P-value is 0.0005*.

b *After Bonferroni correction, the P-value is 0.0015*.

LD of the five SNPs is displayed in Table 3. Owing to the strong LD among four SNPs (rs510432, rs3804338, rs573775, and rs2299863; D' > 0.9), haplotype analysis was performed revealing all five possible haplotypes (ACCG, ACCT, GCCT, GCTT, and GTCT). The most common haplotypes were GCTT (37.1%) and ACCT (34.3%). However, neither the global nor the separate haplotype frequencies showed significant differences between CP and control groups (data not shown).

**Table 3:** The linkage disequilibrium among ATG5 SNPs.

***D'*/*r^2^*^a^**	**rs510432**	**rs3804338**	**rs573775**	**rs2299863**	**rs6568431**
rs510432	–	0.978	0.953	0.826	0.051
rs3804338	0.097	–	0.982	0.928	0.217
rs573775	0.345	0.079	–	–	–
rs2299863	0.065	0.009	0.031	–	–
rs6568431	0.002	0.014	0.010-	0.016	–

a *The standardized D'-values are shown above the diagonal, and the r^2^-values are shown below the diagonal*.

### Subgroup analysis

CP is a multifactorial and complex disease with various clinical and pathological features. Therefore, a subgroup analysis was performed by sex, clinical subtype, birth asphyxia, PROM, PVL, and HIE. Significant differences in both allele and genotype frequencies of rs6568431 were observed between patients with spastic CP (*n* = 468) and controls (*n* = 658; OR = 1.511, 95% CI = 1.251~1.824, *P*_*allele*_ = 8.50e^−005^, *P*_*genotype*_ = 1.57e^−004^), between patients with spastic quadriplegia CP (n = 270) and controls (OR = 1.927, 95% CI = 1.533~2.421, *P*_*allele*_ = 7.35e^−008^, *P*_*genotype*_ = 3.24e^−009^; Table 4), and between patients with maternal PROM (*n* = 68) and controls (OR = 2.041, 95% CI = 1.390~3.000, *P*_*allele*_ = 0.002, *P*_*genotype*_ = 0.00004). The significant difference in allele frequency for rs6568431 between patients with birth asphyxia (*n* = 212) and controls (*P* = 0.011) disappeared after Bonferroni correction. The differences in allele and genotype frequencies were not significant between CP patients with PVL (*n* = 67) or HIE (*n* = 96) and control subjects for any of the five SNPs of *ATG5*.

**Table 4:** Allele and genotype frequencies of ATG5 genetic variants in spastic CP (*n* = 468) and quadriplegia CP (*n* = 270) and controls (*n* = 658).

	**Allele**		***P***	**OR** **(95%CI)**	**Genotype**			***P***	**Hardy-Weinberg equilibrium test**
**rs510432**	A	G			A/A	A/G	G/G		
Spastic CP	252 (0.393)	390 (0.607)	**0.545**	0.940 (0.768~1.150)	64 (0.199)	124 (0.386)	133 (0.414) 76 (0.420)	**0.067**	0.001
Quadriplegia CP	145 (0.401)	217 (0.599)	**0.818**	0.972 (0.761~1.241)	40 (0.221)	65 (0.359)		**0.036**	0.001
controls	414 (0.407)	602 (0.593)			88 (0.173)	238 (0.469)	182 (0.358)		0.502
**rs3804338**	C	T			C/C	C/T	T/T		
Spastic CP	754 (0.875)	108 (0.125)	**0.565**	1.081 (0.830~1.408)	330 (0.766)	94 (0.218)	7 (0.016)	**0.704**	0.918
Quadriplegia CP	427 (0.861)	69 (0.139)	**0.783**	0.958 (0.705~1.301)	186 (0.750)	55 (0.222)	7 (0.028)	**0.348**	0.243
controls	982 (0.866)	152 (0.134)			423 (0.746)	136 (0.240)	8 (0.014)		0.429
**rs573775**	C	T			C/C	C/T	T/T		
Spastic CP	535 (0.648)	379 (0.337)	**0.470**	0.933 (0.772~1.127)	172 (0.416)	191 (0.462)	50 (0.121)	**0.644**	0.786
Quadriplegia CP	305 (0.646)	167 (0.354)	**0.508**	0.927 (0.740~1.161)	102 (0.432)	101 (0.428)	33 (0.140)	**0.717**	0.325
controls	747 (0.663)	379 (0.337)			251 (0.446)	245 (0.435)	67 (0.119)		0.544
**rs2299863**	G	T			G/G	G/T	T/T		
Spastic CP	55 (0.064)	805 (0.936)	**0.816**	1.044 (0.725~1.504)	3 (0.007)	49 (0.114)	378 (0.879)	**0.742**	0.317
Quadriplegia CP	33 (0.067)	461 (0.933)	**0.680**	1.094 (0.713~1.679)	2 (0.008)	29 (0.117)	216 (0.874)	**0.686**	0.359
controls	70 (0.061)	1070 (0.939)			2 (0.004)	66 (0.116)	502 (0.881)		0.914
**rs6568431**	A	C			A/A	A/C	C/C		
Spastic CP	313 (0.409)	453 (0.591)	**1.70e**^−005^a^^	1.511 (1.251~1.824)	78 (0.204)	157 (0.410)	148 (0.386)	**3.14e**^−005^b^^	0.003
Quadriplegia CP	193 (0.468)	219 (0.532)	**1.47e**^−008^c^^	1.927 (1.533~2.421)	60 (0.291)	73 (0.354)	73 (0.354)	**6.47e**^−010^d^^	3.52e^−005^
controls	381 (0.314)	833 (0.686)			63 (0.104)	255 (0.420)	289 (0.476)		0.548

*OR, odds ratio; CI, confidence interval*.

a *After Bonferroni correction, the P-value is 8.50e^−005^*.

b *After Bonferroni correction, the P value is 1.57e^−004^*.

c *After Bonferroni correction, the P-value is 7.35e^−008^*.

d *After Bonferroni correction, the P-value is 3.24e^−009^*.

### Plasma ATG5 quantification

**Figure 1:**
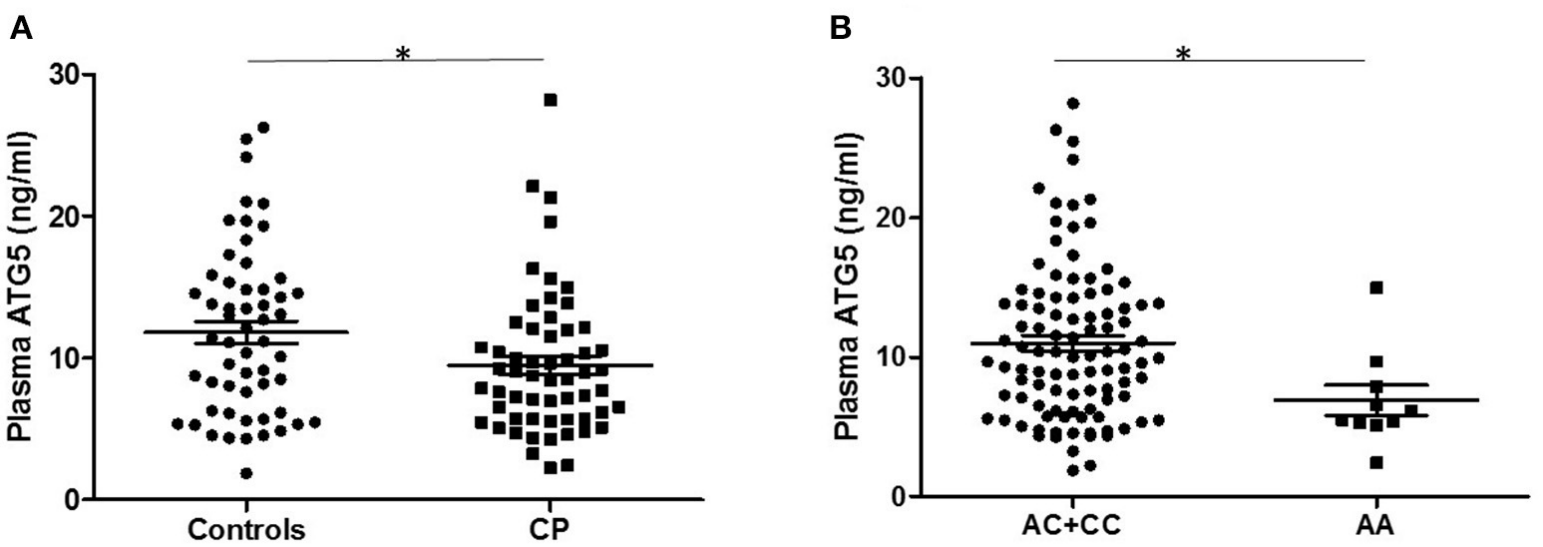
Plasma ATG5 concentrations in patients with controls and CP. **(A)** Distribution of plasma ATG5 concentrations between all patients with controls (56 participants) and CP (58 patients). **(B)** Plasma ATG5 levels in individuals carrying different genotypes of rs6568431 (AC+CC = 100, AA = 10). Each dot represents one individual, and each bar represents the mean value. ^*^P < 0.05; Student's two-tailed *t*-test; CP, cerebral palsy.

To evaluate the effect of ATG5 on the generation and development of CP, 58 patients with CP and 56 controls were selected from the SNP-tested sample for the plasma ATG5 protein assay by ELISA. Mean plasma ATG5 levels were significantly lower in CP patients than that in controls (cases = 9.471 ± 0.658 ng/mL, controls = 11.806 ± 0.785 ng/mL, *P* = 0.024, *P* < 0.05; Figure 1A). Furthermore, plasma ATG5 levels were different between individuals with the AA genotype and those with the AC+CC genotype of rs6568431 (AA = 6.896 ± 1.076 ng/mL, AC+CC = 10.990 ± 0.561 ng/mL, *P* = 0.026, *P* < 0.05; Figure 1B).

## Discussion

The present study is the first to link a genetic variant within the *ATG5* gene to CP. We showed that the rs6568431 SNP of the *ATG5* gene was associated with CP patients. Between 715 cases and 658 controls, the association was still statistically significant after Bonferroni correction (*P*_*allele*_ = 0.0005, *P*_*genotype*_ = 0.0015). Through association analysis between SNPs and subgroups, only rs6568431 was associated with spastic, spastic quadriplegia, and PROM subgroups. The strongest correlation was found in spastic quadriplegia CP, suggesting that ATG5 might have a more important influence in severe types of CP. Furthermore, the observation of lower plasma ATG5 levels in CP patients and in individuals with the AA genotype of rs6568431 indicates that the variant of ATG5 confirmed in CP patients may change the level of Atg5 protein and interfere with the activity of autophagy, thereby acting as a risk factor for CP onset.

Till now, about 35 *ATG* genes have been confirmed. Their functions are mostly related to autophagosome formation, and are conserved from yeast to humans (Zhou and Zhang, 2012). Associations between *ATG* SNPs and some diseases have been identified in multiple populations; e.g., the associations between polymorphisms in *ATG16L1* with inflammatory Crohn disease (Hampe et al., 2007), palmoplantar pustulosis (Douroudis et al., 2011), and psoriasis vulgaris (Douroudis et al., 2012), as well as polymorphisms in *ATG10* with breast cancer (Qin et al., 2013) and Vogt–Koyanagi–Harada syndrome (Zheng et al., 2015). These studies indicate that autophagy has multiple effects in the development of diseases.

A variety of studies have showed that variants of *ATG5* gene are related to certain diseases of the immune system and neurodegenerative diseases. Genome-wide association studies of SLE in Chinese Han and European populations reported that the rs6568431 SNP of *ATG5* was significantly associated with SLE (Graham et al., 2009; Han et al., 2009). A previous study discussed an association of rs573775 with Behçet's disease (BD) in the Chinese southern Han population, and the TT genotype of rs573775 avoided developing BD (Zheng et al., 2015). Chen et al. (2013) studied genetic variants of the *ATG5* gene promoter (e.g., rs510432) in sporadic PD. Until now, no study has shown an association between these SNPs and CP. Our study is the first to focus on the association between SNPs in *ATG5* and CP and to show a significant association between *ATG5* rs6568431 and CP. According to our findings, the presence of the rs6568431 A allele of the *ATG5* gene significantly increased the risk of CP.

Association of *ATG5* with different diseases suggests a pleiotropic role of *ATG5* in many physiological processes and a potentially shared pathomechanism underlying these diseases, which include neurodegenerative diseases, autoimmune diseases, and inflammatory diseases. These findings also indicate that a disruption of immunological homeostasis might be implicated in the pathophysiology of CP. The reviewed data showed that immune regulatory cells are potentially involved in the chronicity of inborn brain damage (Lisovska et al., 2016). The genotype–phenotype correlations of ATG5 are complicated, and *ATG5* could also be related to the variability of cell vulnerability in different tissues at different ages and different environmental factors.

Subgroup analysis based on clinical classification can effectively reduce the effect of phenotypic heterogeneity on association analysis in patients with CP. The present study showed for the first time that the association between the *ATG5* polymorphism at rs6568431 and CP is the strongest in spastic quadriplegia, the most severe type of CP (Caselli et al., 2017). Our results suggest that the correlation between *ATG5* variation and CP is more robust in patients with more serious brain damage and disease phenotype.

To explore the correlation between *ATG5* gene variants and ATG5 protein expression, we measured ATG5 levels directly. Interestingly, plasma ATG5 levels were lower in patients with CP, and different genotypes of rs6568431 showed different levels of ATG5. This finding is similar to those of BD, in which functional studies showed that the *ATG5* gene variant was correlative with ATG5 expression (Zheng et al., 2015). We speculate that genetic variation of *ATG5* might change (downregulate) ATG5 protein levels and alter autophagic activities during the pathogenic process of CP. The AA genotype of rs6568431 may be a risk factor of CP, though this needs to be confirmed with a larger sample size.

The contradictory roles of autophagy are often viewed as confusing. In particular, the regulation of autophagy in the nervous system remains largely unknown. Inhibition of autophagy could be a new strategy to prevent ischemic brain injury. Previous studies have shown that the inhibition of autophagy reduces brain damage after cerebral ischemia (Xing et al., 2012; Xie et al., 2016), and during subsequent reperfusion, the protective role of autophagy is probably caused by mitophagy-related mitochondrial clearance and inhibition of downstream apoptosis (Zhang et al., 2013). Furthermore, *in vivo* studies in mice deficient in *Atg5* showed that autophagy may play a protective role against the development of many neurological degenerative diseases. These findings suggest that in the adult brain, ATG5 may function via the autophagy pathway to regulate the survival of neural progenitor cells during their development into new functional neurons (Xi et al., 2016). ATG5-dependent autophagy, which is thought to constantly remove diffuse cytosolic proteins, is very important to prevent abnormal protein accumulation, because these abnormal proteins could disrupt neural function and ultimately lead to neurological degeneration (Hara et al., 2006). In addition, ATG5 may play an important role in preserving axon morphology and membrane structures. Axonal swelling resulting from a lack of ATG5 function is followed by progressive neurodegeneration in mammalian neurons (Nishiyama et al., 2007).

ATG5 may also play an important role at birth, when neonates adapt to the suddenly interrupted trans-placental nutrient supply by inducing autophagy. This is supported by a study showing that amino acid levels in plasma and tissues were reduced and signs of energy depletion were obvious in *Atg5*-deficient neonatal mice (Kuma et al., 2004). Our finding that mean plasma ATG5 levels were significantly lower in patients with CP seems to support the conclusions of the above studies, namely that ATG5-dependent autophagy has a protective role against the development of CP. However, it is still unknown if the decreased ATG5 level was due to the feedback inhibition of autophagy activity or not, because previous studies showed that autophagy activity was increased in the acute phase of neonatal brain injury (Ginet et al., 2014; Xie et al., 2016). There is no report yet of autophagy activity in the chronic phase of brain injury such as cerebral palsy. For clarifying this, further investigations monitoring the dynamic changes of autophagy activity in neonatal brain injury are needed.

How the *ATG5* gene protects against the development of CP is still unclear. In the current study, we analyzed five polymorphisms of *ATG5* gene in CP patients and ATG5 protein levels in plasma, and found that variants of the *ATG5* gene and low levels of ATG5 protein were significantly associated with CP. Our results showed that patients with CP displayed decreased expression of ATG5, which may be associated with fetal or infant brain injury. Thus, we hypothesize that the variant of the *ATG5* gene identified in our study may be associated with downregulation of ATG5 protein levels, and the reduced ATG5 protein expression might result in a lower level of autophagosome formation. Therefore, the protective role of ATG5-dependent autophagy against fetal or infant brain injury may weaken and ultimately lead to the development of CP. Moreover, the autophagy-independent functions of ATG5 have been described (Jounai et al., 2007; Zhao et al., 2008; Tal et al., 2009; Zhou and Zhang, 2012; Dipak et al., 2013; Jacqueline et al., 2015); ATG5 may still be involved in the pathogenesis of CP through an autophagy-independent mechanism, despite being an autophagy gene. Further studies are needed to test this hypothesis and examine the role of epigenetic mechanisms of ATG5 in CP.

In conclusion, this study is the first to analyze the association of SNPs in *ATG5* and plasma ATG5 levels with the development of CP in the Chinese population. We found that a variant of the *ATG5* gene and low levels of ATG5 protein were significantly associated with CP. The variant may affect the expression of ATG5 and alter the activity of autophagy, which may contribute to CP as a risk factor. *ATG5* gene polymorphisms might therefore be related to the development of CP. Additional studies are necessary to further elucidate the biological functions of autophagy in CP and to investigate potential autophagy-related therapy for patients with this disease.

## Author contributions

JX and LX: performed experiments, analyzed data, and wrote the manuscript; DB, JS, and YW: performed experiments and analyzed data; JS, QS, DZ, CM, CG, XZ, YS, and LX: provided samples; JD, XW, and LZ: analyzed the data; CZ and QX: designed the study and revised the manuscript. All authors read and approved the final manuscript.

### Conflict of interest statement

The authors declare that the research was conducted in the absence of any commercial or financial relationships that could be construed as a potential conflict of interest.
